# Anomaly detection using machine learning and adopted digital twin concepts in radio environments

**DOI:** 10.1038/s41598-025-02759-5

**Published:** 2025-05-26

**Authors:** Mohamed Hussien Moharam, Omar Hany, Ahmed Hany, Amenah Mahmoud, Mariam Mohamed, Sohila Saeed

**Affiliations:** Electronics and Communications Engineering Department, Misr University for Science and Technology, Giza, Egypt

**Keywords:** Digital twin (DT), Received signal strength Indicator (RSSI), Support vector machine (SVM), Logistic regression (LR), Random forest (RF), Electrical and electronic engineering, Scientific data, Characterization and analytical techniques

## Abstract

Reliable and secure wireless communication is essential in Industry 4.0. This work presents an anomaly detection framework using Digital Twin (DT) technology to simulate and monitor dynamic radio environments. By modeling network conditions and attack scenarios, the DT enables accurate identification of anomalies, particularly security threats. This study integrates machine learning with anomaly detection frameworks to enhance wireless network security. The proposed approach creates a virtual representation of the wireless environment, enabling accurate identification of anomalies and security threats. To validate the effectiveness of this framework, multiple machine learning algorithms based on traditional classifiers which are compared for their ability to detect anomalies, particularly jamming attacks. XGBoost achieved the highest accuracy (0.99) and perfect detection (1.00) of normal traffic and signal drift, outperforming Random Forest (0.98), Support Vector Machine (0.97), Logistic Regression (0.93), and K Nearest Neighbors (0.81). These results highlight XGBoost as a reliable solution for wireless network security. This work contributes to ongoing research on the integration of DT for comprehensive wireless network monitoring, emphasizing their potential to improve anomaly detection and resilience in next-generation communication systems.

## Introduction

Wireless networks are a central component for contemporary industrial structures, particularly following the advent of Industry 4.0, where security and reliability for these networks become extremely important. While their sophistication rises, the more difficult it is to guard them against disruptions, abnormalities, and cyber attacks. Identifying anomalies in radio environments is key to ensuring the security and efficiency of these systems after developing new security vulnerabilities to Industrial Control Systems (ICS)^1^.

### Motivations

This research introduces a novel approach to anomaly detection in wireless networks by leveraging DT technology^2^. While drawing inspiration from the study “Digital Twin of the Radio Environment: A Novel Approach for Anomaly Detection in Wireless Networks,” our work depends entirely on custom generated data and proprietary code^3,4^. Unlike previous studies, we developed our dataset independently, ensuring it is original and matches our research goal.

### Contribution

Our dataset, consisting of over 20,000 samples generated within an environment size of 40 m x 40 m, captures important network parameters such as RSSI (Received Signal Strength Indicator), Path Loss, SNR (Signal to Noise Ratio), attack types, severity, and potential countermeasures^5^. Labeled anomaly classes include “Normal,” “Signal Drift,” “Multipath Effect,” and “Localization Inaccuracy.” Every element of this dataset reflects our unique methodology and computational framework, emphasizing the uniqueness and authenticity of our contribution.

Through a detailed data generation process, we identified key relationships between parameters such as RSSI and Path Loss, proving these as reliable indicators for detecting network anomalies and attacks^6,7^. The dataset enables effective training and evaluation of machine learning algorithms to achieve high accuracy in anomaly detection and classification^8^. Results validate the effectiveness of these models in discovering anomalies based on their type and severity, highlighting the practical implications of our approach in real world network environments^9^.

Anomaly detection in cellular networks is crucial for identifying cyber security threats and performance issues, particularly in complex radio environments in now days industry. Our approach utilizes machine learning algorithms, including KNN, RF, XGBoost, LR, and SVM, to categorize anomalies based on key parameters such as RSSI, Path Loss, SNR, and Localization Error. By creating a Python based framework, we implemented a threshold based detection system inspired by the concept of Signal Intelligence, allowing us to distinguish between normal and anomalous network behavior. Where, the detection of multipath effects (classified as Anomaly Class 2) is done by filtering cases where RSSI drops under a threshold while SNR remains high, indicating signal reflections. This is visualized through scatter plots that highlight the affected signals based on distance and path loss. Additionally, our research integrates a security layer that maps detected anomalies to potential cyber threats, supporting the idea that even seemingly benign signal variations may correspond to security incidents. This approach enhances real time anomaly detection and strengthens the security posture of wireless networks by correlating radio anomalies with possible attack scenarios.

A key advancement of this work lies in the scalability and adaptability of our solution. By using our own dataset and custom code, the research is not constrained by the limitations of already existing data or methods, enabling more detailed exploration of complex scenarios. Additionally, the dataset’s structure supports various wireless network configurations, making it a flexible tool for ongoing research and development. This flexibility ensures that the framework can be adjusted to address emerging threats and dynamic network conditions.

This research highlights the critical role of innovation in data generation and system modeling to enhance the strength of wireless communication systems^10^. By providing an entirely original dataset and using DT technology, our study contributes a significant advancement to the field, offering a scalable solution for improving security and performance in wireless networks. We compare our proposed algorithm with the one used in Reference^11^. While the reference uses a specific algorithm, our method uses a sample based approach, which is less complex yet more effective. This comparative analysis strengthens our research by showing the advantages of our approach over the reference method. The methodology and findings presented here provide a strong foundation for future work aimed at strengthening wireless communication systems in an era of rapid technological evolution.

The work is divided into five sections. In Sect. 2, introduces existing work on anomaly detection in DT wireless networks and the related challenges. In Sect. 3, it includes details of dataset generation, anomaly classification, security integration. In Sect. 4, provides a comprehensive analysis of the performance and effectiveness of various machine learning models applied to anomaly detection and its results. In Sect. 5, highlighting and concluding the most important learnings from this work.

## Related work

The references mentioned in this section form a complete view of how research in the challenge of ensuring stability in wireless communication networks, especially with the growth of interconnected systems in Industry 4.0, where disruptions, such as jamming, can have severe effects. Several approaches have been proposed to address network stability and anomaly detection in the context of wireless communication^12,13^.

Early work in anomaly detection focused on detecting interference and jamming using traditional methods, including signal strength measurements, error rates, and signal to noise ratios. A common approach for identifying anomalies, such as jamming, involves the analysis of spectrograms, where machine learning (ML) techniques are employed to classify signals as either normal or anomalous^14,15^. Supervised learning models have been explored, where training data consisting of both normal and abnormal spectrograms are used to train classifiers the characteristics of various interference scenarios^16,17^. While effective, supervised methods face challenges in detecting unseen anomaly types during operation, which limits their flexibility in real world scenarios^18,19^.

To address these limitations, unsupervised learning techniques have gained attention. For example, autoencoders have been utilized to rebuild spectrograms, with anomalies identified as variance from the expected rebuilding^20,21^. Another unsupervised approach involves predictive models that estimate expected signal behaviors, highlighting any variations as potential anomalies^22,23^. Additionally, some studies have focused on monitoring bit error rates and packet error rates to detect interference or failures in wireless networks^24,25^.

Although there have been advances in these anomaly detection techniques, most existing methods fail to incorporate situational information about the network environment^26^. This includes factors like transmitter positions, physical obstacles, and signal propagation patterns^27,28^. While DTs have been widely utilized in industries such as manufacturing, their application to telecommunications networks is still in the research phase^29,30^. Several studies have begun to explore the potential of using DTs to model wireless environments for the purpose of anomaly detection^31^.

The integration of Combined Communications and Sensing (JCAS) and accurate indoor positioning systems, expected in future 6G networks, provides a promising foundation for creating a DT of the radio environment^32^. These technologies enable the precise tracking of transmitters and the propagation of signals, allowing for better estimation of expected signal strengths^33^. Propagation models, such as ray tracing, can then be used to predict expected received signal strengths (RSS) at various sensing points across the network, providing a standard against which real time measurements can be compared^34^.

The advancement of this approach exists in its ability to integrate relevant information, such as the known locations and characteristics of regular transmitters, into the anomaly detection process^35^. The proposed method compares the expected RSS values from the DT with the actual measurements collected by distributed sensing units (SUs). Variations from the expected values indicate potential anomalies, such as jamming events^36^. This approach does not require previous knowledge of jammer characteristics and can detect a wide range of anomalies based only on normal operational data. This capability marks an important improvement over traditional methods that depend only on spectrograms or signal characteristics^37–39^.

In summary, while much of the existing research focuses on machine learning based anomaly detection and spectrogram analysis, the integration of a DT of the radio environment, Utilizing the advanced capabilities of 6G networks, represents a promising direction for future wireless network stability. The proposed method provides a novel framework for anomaly detection, opening the way for more flexible and strong networks suited to handle unexpected disruptions, such as jamming.

## Methodology

Our methodology begins with creating a dataset using system parameters referenced in Reference Paper^11^. This data generation was accomplished using our Python framework, which consists of six dedicated code modules. Each module handles a specific part of data generation, cleaning, and grouping.

The system structure assumes a 40 × 40 m radio environment free of obstacles. Within this area, there are 10 transmitters and 0 or 1 jammer, each operating at the same power level as specified in the table below. A key parameter in our study is the RSSI, which reflects the strength of signals received by the SUs from either the TXs or jammers. All these components are connected to a control unit running our framework.

Anomalies in the radio environment are identified through examination of localization data, RSSI, and SNR values. This process identifies active transmitters and their effect on the 25 SUs spread across the area. This setup enables signal intelligence analysis for detecting anomalies in the radio environment based on predefined threshold values. To extend anomaly detection into a security centric framework, anomalies were mapped to specific cyber threats. This mapping process follows:

1. Feature Alignment: The extracted features (RSSI, SNR, Path Loss, Localization Error, etc.) were mapped to known attack behaviors.
2. Threat Classification: Each detected anomaly was categorized under a predefined security attack type.
3. Severity Assessment: Attacks were assigned severity levels (High/Medium) based on potential system disruption.
4. Countermeasure Mapping: Each attack was linked to a security mitigation technique, ensuring real time defense adaptation.

### Dataset generation

We generated a comprehensive anomaly detection dataset with over 20,000 samples using python code. The dataset is based on parameters and environmental characteristics like those in^3^. Particularly, we modeled network conditions within a 40 m × 40 m radio environment. Key parameters were derived from Table 1.

**Fig. 1:**
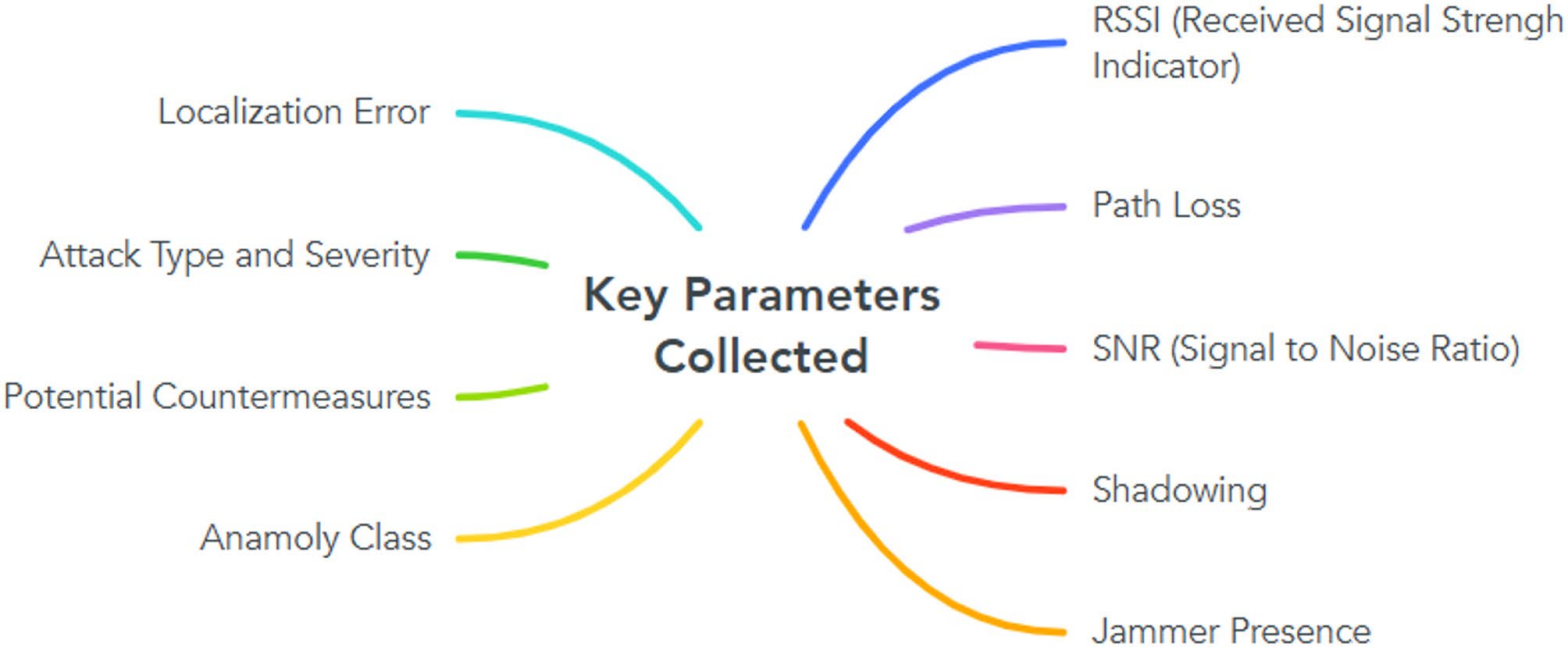
Key parameters collected.

Figure 1 shows parameters that were selected based on their relevance to detecting anomalies in wireless communication environments. The dataset was divided into different anomaly classes: “Normal,” “Signal Drift,” “Multipath Effect,” “Localization Inaccuracy,” “HShadLev,” and “LShadLev.” Each sample was labeled with its respective anomaly class, which helped in training the machine learning models and evaluating the effectiveness of anomaly detection.

**Table 1:** Our proposed system parameters.

Parameter	Value
Area size	40 m x 40 m
Carrier Frequency $\:{f}_{c}$	3.7 GHz
Transmit power of regular transmitters $\:{P}_{\left(tx,reg\right)}$	20 dBm
Transmit power of jammers $\:{P}_{\left(tx,jam\right)}$	20 dBm
Path loss exponent α	2
Path loss offset $\:{L}_{o}$	-147.55 dB
Shadowing standard deviation (positioning error)	1.33 m

### Anomaly detection and classification

**Fig. 2:**
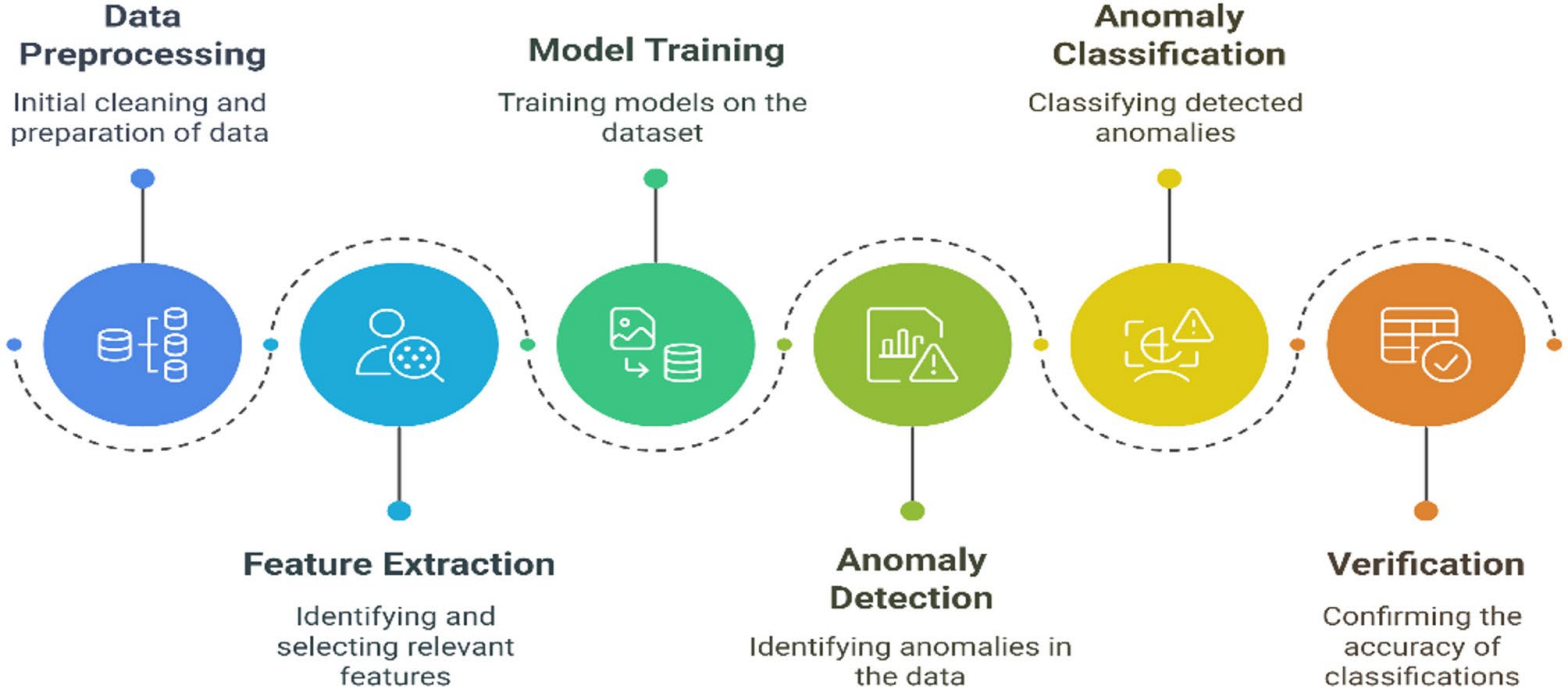
Anomaly detection and classification.

Figure 2 shows the systematic approach used in anomaly detection and classification under the domain of machine learning; the detailed workflow starting from data preprocessing to validation of the model results is presented. The first step, Data Preprocessing, includes cleansing and normalization of the dataset in order to bring consistency and minimize noise in the data; this is crucial for training the model accurately. After preprocessing, feature extraction is realized by identifying and extracting pertinent parameters: RSSI, Path Loss, SNR, and attack metrics that are relevant to the anomaly detection process. These features are instrumental in capturing the patterns associated with both normal and anomalous data.

The Model Training phase applies machine learning algorithms to learn relationships between extracted features and anomalies, using labeled datasets for supervised training. This step establishes the foundation for resilient detection and classification mechanisms. By Using 4 steps in each algorithm “Randomized cv search, Hyperparameter tuning, Permutation importance, Extraction Weights”.

Following training, Anomaly Detection is carried out on both the preprocessed dataset and real time inputs. This step target is to detect deviations indicative of potential anomalies, including signal drift, jamming, and multipath effects.

Detected anomalies are then categorized into predetermined classes during the Anomaly classification phase. Classes such as (Normal, Signal Drift, Multipath Effect) making sure that detected anomalies are accurately categorized according to its characteristics.

Finally, the Verification step evaluates the accuracy of classifications to verify the performance of the machine learning model. Figure 2 represents the impact of the models, estimated according to their accuracy in detecting and classifying anomalies in both the generated and real time datasets.

### Security layer integration

Table 2 outlines information according to different types of attacks commonly experienced in wireless communication systems. Each attack is represented in detail, highlighting it’s mechanism and potential risks. The table also represents a severity Level column, which categorizes attacks as High or Medium depending on the damage they can cause to the network and system integrity. The potential countermeasure column suggests defensive strategies customized for each attack type. For example, jamming Attacks require adaptive frequency hopping to avoid interference, while MITM can be highlighted by using strong encryption and validation techniques. Similarly, replay Attacks and eavesdropping attacks take advantage of signal monitoring and encryption improvements. This representation aligns perfectly into the anomaly detection framework, particularly the security layer, to offer practical insights. By categorizing attacks and connecting their severities to corresponding countermeasures, this table enables quick reference and improve the entire defense strategy of the system.

**Table 2:** Attack types, severity levels, and suggested countermeasures.

Attack Type	Description	Severity	Potential Countermeasure
Jamming Attack	Overwhelms the communication channel with noise or signals, preventing legitimate communication.	High	Adaptive frequency hopping or spread spectrum techniques.
Man-in-the-Middle Attack	Exploits signal reflections or delays to intercept or alter communication. Risks include data theft.	High	Implement signal encryption and route validation protocols.
Replay attack	Captures legitimate signals and retransmits them to disrupt or confuse the system.	Medium	Increase signal filtering and monitor for abnormal variations.
Eavesdroppig attack	Exploits weak signals to intercept communication, leading to data leakage and loss of confidentiality	Medium	Enhance encryption and monitor signal consistency.

### Model workflow

**Fig. 3:**
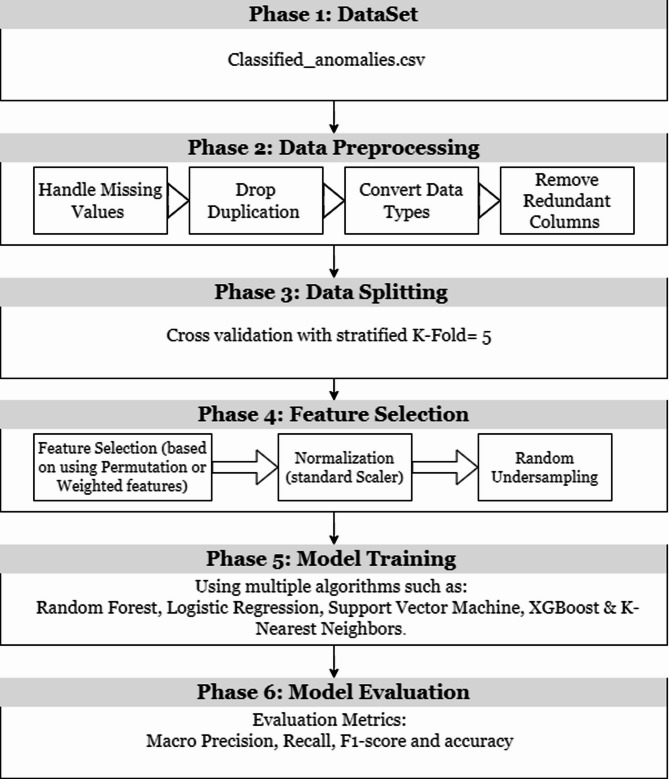
Overview of model architecture.

The workflow in Fig. 3 present an overview of the methodology for enhancing a machine learning according to wireless Network anomaly Detection System using the Classified anomalies dataset. The process consists of the following stages:

1. Gathering Data: use the Classified anomalies dataset, consisting of 20000entries and 18 features, representing various types of cyber-attacks.
2. Data Preprocessing: categorize data quality issues by handling missing or unreliable values, removing duplicate entries, converting data types, and eliminating redundant columns to improve data efficiency.
3. Feature Selection: Resolve class imbalance through random undersampling of the majority class, normalize data using standard scaling to ensure they are on the same scale, and select the most relevant features using techniques such as Recursive Feature Elimination (RFE) & correlation analysis.
4. Data Splitting: use a segmented K fold cross validation with five folds to split the data while maintaining class spread for precise model validation.
5. Model Training: train and test models by applying different algorithms as (RF, LR, SVM, XGBoost, KNN).

For each algorithm, consistently perform optimize Hyperparameters using randomized search, Fine tune model parameters for better performance, assess feature importance analysis during training, and incorporate class weights to handle class imbalance effectively.

6. Model Evaluation: Assess overall model performance using metrics such as Weighted and Macro Precision, Recall, Accuracy, and F1 Score evaluation.

In the following subsections, every step is explained in detail.

### Data leakage

To guarantee the integrity of the anomaly detection framework, there were stringent measures put in place to avoid leakage of data during processing. Key processes such as data normalization, feature selection, under sampling, and hyperparameter tuning (using randomized search) were performed exclusively on the training set to ensure that no information from the test set influenced these procedures. This separation maintained the integrity of the model evaluation process and provided unbiased results. Furthermore, we utilized repeated stratified K fold cross validation to evaluate the models. This method ensured even distribution of data across all folds, preserving the balance of anomaly classes and keeping the training and testing datasets independent. By following this approach precisely, we reduced the possibility of unintentional data leakage, preserving the accuracy of performance metrics and improving the model’s ability to perform well on new, unseen data.

#### Dataset columns

- Sensing Unit (SU): Identifies the distributed sensing unit in charge for gathering data.
- Source Type: refers to the type of source as(jammer or transmitter), that contributes to communication signals.
- Distance (m): Distance between the source and the SU in meters.
- Path Loss (dB): Signal power loss as it propagates through space.
- RSSI (dBm): Received Signal Strength Indicator, representing the power of signal at the receiver.
- SNR (dB): Signal to Noise Ratio, Ratio between signal and noise.
- Shadowing: models for signal attenuation caused by environmental shadowing effects.
- Jammer Present: A binary indicator used for indicating a jamming attack.
- Localization Error: indicates how inaccurately the source location was determined. • No Anomaly Detected: Indicates whether the observation corresponds to normal network behavior.
- Other Anomalies: Includes other network irregularities not classified under specific attacks.
- Attack Type: specify the type of attack as shown in Table 2.
- Attack Severity: attack severity is represented categorically.
- Potential Countermeasure: Suggested mitigation strategies for each attack type.
- Description: A brief explanation of the observed anomaly or attack. Anomaly Class: A numeric classification of anomalies ranging from 0 (Normal) to various types of network disruptions (non zero) as shown in Table 3.

**Fig. 4:**
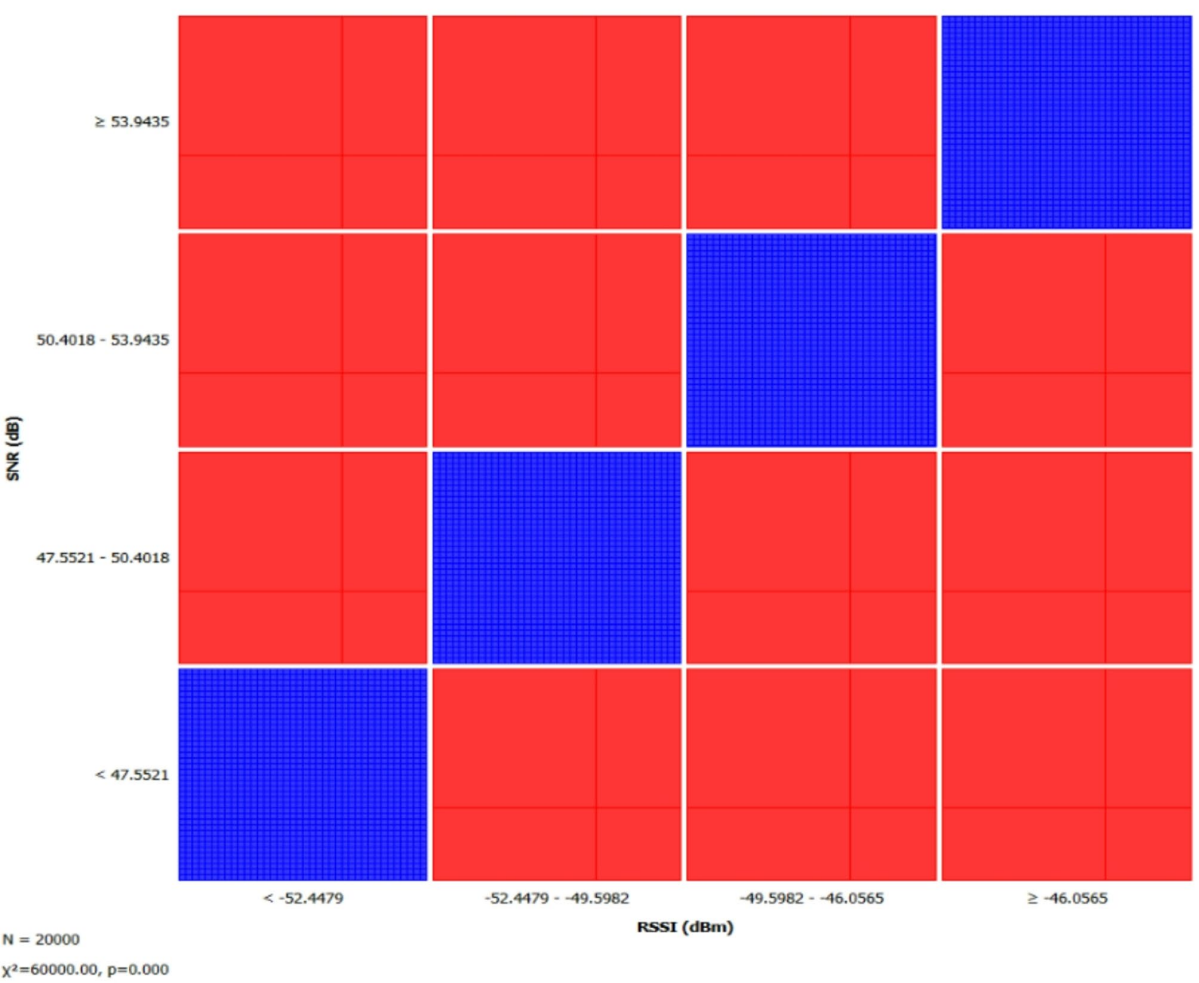
Sieve diagram showing the relationship between RSSI (dBm) and SNR.

Figure 4 visualizes the correlation between RSSI and SNR using a sieve diagram, where the x axis resembles RSSI into four intervals and the y axis divides SNR into four ranges. Red regions indicate higher than expected occurrences, likely due to stable signal conditions or reduced interference, while blue regions represent lower than expected occurrences, suggesting disruptions caused by environmental factors, hardware limitations, or interference. The diagonal pattern of blue regions implies that mid range RSSI values do not consistently align with moderate SNR, meaning that as RSSI improves, SNR does not always increase proportionally. The chi square statistic (χ^2^ = 60,000, *p* = 0.000) confirms a strong correlation, revealing that signal strength and quality deviate from a uniform distribution. This pattern provides insights into signal propagation characteristics and potential interference effects within the dataset.

#### Data generation

Table 3 outlines a classification framework specifically designed to capture key anomalies and environmental factors that affect the performance of wireless networks. Six classes in this framework, each representing different network behaviors or challenges. The dataset created using the parameters in Table (1) to resemble real world conditions, is an essential resource for evaluating machine learning algorithms and anomaly detection models, offering a robust foundation for the development of more resilient and efficient cellular systems.

1. Class 0 Normal: This class represents the initial state of the network, where no anomalies are detected, and optimal functionality has occurred. It serves as the baseline category in comparison with other anomalous conditions.
2. Class 1 Signal Drift: Signal drift refers to changes in metrics like RSSI or SNR, often due to natural network fluctuations or user behavior, without external interference. While not immediately alarming, it can indicate issues like declining hardware or environmental changes.
3. Class 2 Multipath Effect: The multipath effect happens when signals scatter along different paths, causing fluctuations in strength and quality due to interference. Class 2 addresses these distortions, which can lead to unstable connections and impact network performance. This is particularly an issue in areas with complex obstacles.
4. Class 3 Class 3 focuses on errors in establishing the exact location of a source or sensing unit, caused by environmental factors or system limitations. These errors can lead to inaccurate position data, affecting network resource allocation. These errors are essential for maintaining system reliability, particularly in environments mobility which is very important to select appropriate location.
5. Class 4 HShadLev (High Shadowing Levels): It refers to situations where environmental obstacles, such as buildings, cause significant signal attenuation. This results in critical degradation of the signal, often leading to weak or nearly unavailable connections.
6. Class 5 LShadLev (Low Shadowing Levels): This class represents conditions with minimal signal attenuation, where environmental factors have little impact on signal propagation, ensuring more stable communication. The dataset, created to simulate real world conditions, includes these labeled classes to aid in developing and testing machine learning models for anomaly detection. By modeling factors like shadowing and path loss, it offers a strong foundation for advancing wireless network security and resilience.

**Table 3:** Class mapping.

Class number	Class type	Class description
0	Normal	No anomalies detected; network functions optimally.
1	Signal Drift	Gradual changes in signal characteristics without external interference.
2	Multipath Effect	Signal distortion caused by multiple propagation paths.
3	Localization Inaccuracy	Errors in determining the position of a source or sensing unit.
4	HShadLev	Represents significant environmental attenuation, causing severe signal degradation and weak connections, particularly in urban areas.
5	LShadLev	Represents conditions with minimal environmental attenuation, ensuring stable signal propagation and communication.

### Dataset specifications

A dataset of 20,000 samples was created to support strong anomaly detection in wireless networks. It was generated using tailored built scripts, inspired by studies, and simulated within a 40 m x 40 m area under realistic environmental conditions according to Table 1.

### Data type optimization

To improve efficiency, data types were optimized for lowered memory usage, faster processing, and scalability:

- Integer Columns: Discrete values like Source Index and Anomaly Class stored as integers.
- Floating Point Columns: features like RSSI and SNR stored as float32 for fair precision and reduced memory.
- Categorical Data: to save memory string values are transformed to categorical types.
- Binary Columns: to save storage space, boolean data is stored as binary.
- String Columns: Unique descriptive entries kept as strings where compression is unnecessary.

The Benefits of These optimizations decreased memory usage, improved computational performance, and enhanced scalability, supporting efficient anomaly detection and scalability for future expansions.

### Data pre‑processing

The preprocessing involved the following steps:

- Handling Missing Values:

1. Missing data in columns such as Shadowing and Localization Error was imputed using the mean or median value, depending on the distribution of the data.
2. Records with excessive missing values were discarded to maintain dataset integrity.

- Feature Scaling:

3. Continuous variables such as distance (m), Path Loss (dB), RSSI (dBm), and SNR (dB) were standardized using z score normalization to guarantee all features contributed equally to the machine learning models. This reduced the bias proposed by features with highest magnitudes.

- Outlier Detection and Removal:

4. Outliers in numerical columns were detected using the Interquartile Range (IQR) method. Records with values beyond 1.5 times the IQR were flagged for removal.
5. For anomaly related attributes, extreme outliers were retained if they represented valid anomalies, as they were crucial for model training.

- Encoding Categorical Variables:

6. Columns like Source Type, Attack Type, Attack Severity, and Potential Countermeasure were converted into numerical representations using one hot encoding or label encoding, depending on the column’s characteristics.
7. The Anomaly Class column, which represents the target variable, was encoded as an integer for classification tasks.

- Data Augmentation:

8. Noise was added to features like RSSI (dBm) and SNR (dB) to simulate real world variations and improve the robustness of the models.
9. Rotations and translations were applied to spatial parameters like Distance (m) to mimic different environmental conditions.

- Splitting the Dataset:

10. The dataset was structured for model evaluation using stratified 5 fold cross validation, confirming that class spreading was preserved across all folds.
11. This approach allowed the dataset to be systematically divided into five subsets, where each fold served as a validation set once while the remaining four folds were used for training, providing a robust evaluation framework.
12. Feature importance was assessed using correlation analysis technique. Redundant features were rejected to streamline the dataset and enhance model performance.

- Noise Reduction:

13. Signal related columns like RSSI in dBm and SNR in dB were smoothed using moving average filters to decrease noise without compromising the integrity of the data.

**Fig. 5:**
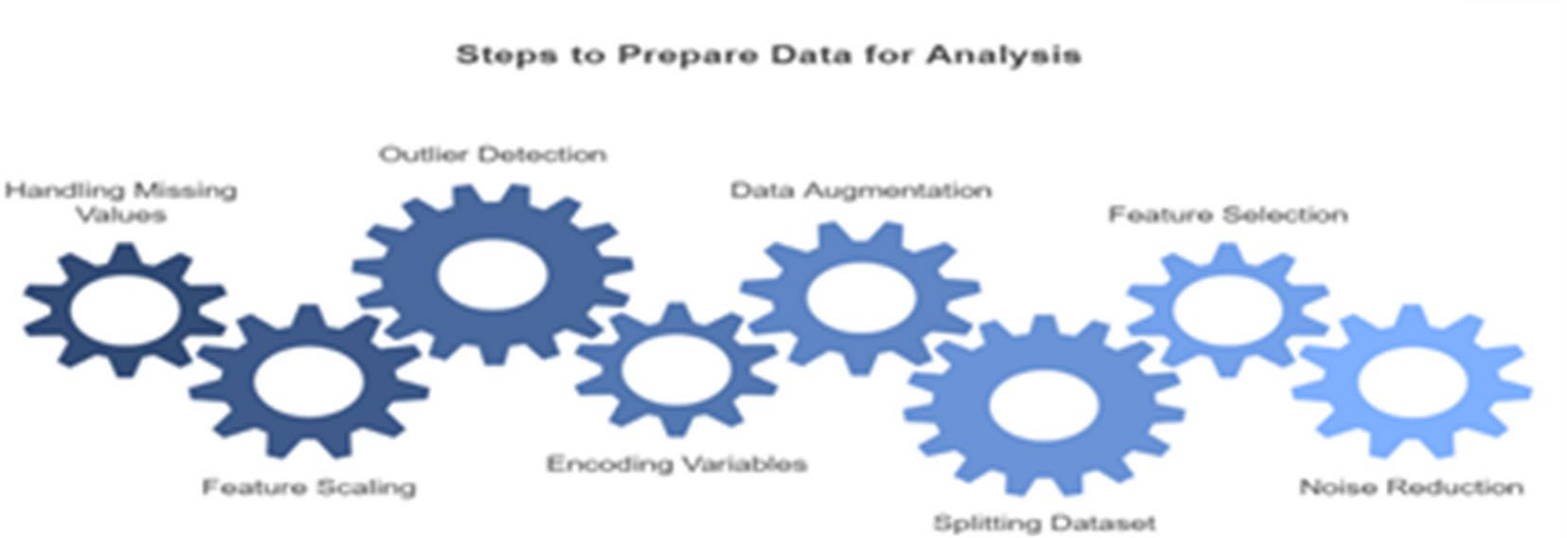
Anomaly detection data preparation.

Figure 5 represents the critical data preprocessing steps implemented to prepare the dataset for machine learning tasks. By ensuring the dataset is clean, consistent, and suitable for anomaly detection, thereby enhancing model accuracy and reliability. Initially, missing values in numerical and categorical columns were handled using imputation techniques or removal when excessive gaps were identified. Continuous variables including Distance, Path Loss, RSSI, and SNR, were normalized using z score normalization to remove biases due to changing feature magnitudes. Outliers were discovered using the Interquartile Range technique, with exceptions made for anomaly specific features that served as valid inputs for model training. Categorical features, including Source Type and Attack Severity, were numerically encoded using one hot or label encoding, while the target variable Anomaly Class was modified for classification. Data augmentation techniques, such as introducing noise to RSSI and SNR and preforming spatial rotations and translations, were used to imitate real world variations and environmental conditions. The dataset was prepared for model assessment using stratified five fold cross validation, with class distributions retained across all folds. Feature selection was performed using Recursive Feature Elimination (RFE) and correlation analysis to eliminate duplicates features and improve model preformance. Lastly, noise reduction technologies, such as moving average filters, were used on signal based features to improve data quality while maintaining integrity.

#### Missing values and unreliable values

Continuous variables, such as Distance (m) and RSSI (dBm), were filled using the mean or median values to preserve the dataset’s statistical properties. Categorical variables, such as Source Type and Attack Type, were imputed with the most frequent category. Records with a high percentage of missing values across multiple features were discarded to maintain overall dataset quality.

#### Correlation and dropping redundant features

Redundant categorical variables were evaluated using Cramér’s V statistic to determine their correlation, ensuring that the retained features offered unique insights. This process reduced the dataset’s dimensionality, improved computational efficiency, and enhanced model performance by eliminating noise and redundancy.

#### Data duplicates

In our dataset, we performed a full analysis to detect and eliminate duplicate rows, considering all feature columns as criteria for duplication. Duplicate records were detected when the values in all columns matched exactly across rows. After identifying these duplicates, they were removed to avoid affecting model training with redundant information.

The process was further refined to account for partial duplicates cases where most features were identical except for small differences in non critical fields, such as comments or descriptions. In these cases, domain expertise was applied to review the importance of retaining or merging these entries.

By resolving data duplication, we ensured the uniqueness and authenticity of the dataset, which contributes to balanced model training and a more accurate evaluation of anomaly detection algorithms. This step also helped improve computational efficiency by reducing the overall dataset size without reducing its quality.

### Data grouping and data sampling

The gain of grouping was important for feature engineering and model validation steps, as it enabled us to maintain the essential structure of the data while ensuring balanced representation across different categories during sampling.

Given the large size of the dataset (20,000 samples), data sampling was applied to create balanced and manageable groups for model training, testing, and validation. Sampling techniques were employed to address the following objectives:

- Balancing Class Distribution.
- Reducing Computational Overhead.
- Addressing Specific Use Cases.

### Hyperparameter fine‑tuning

In the field of DT Wireless Networks, effective anomaly detection is essential for maintaining network integrity and performance. To enhance the performance of various machine learning models used for this purpose, thorough hyperparameter tuning was methodically implemented. This process involved adjusting key parameters for each algorithm to optimize accuracy, precision, recall, and overall stability. Techniques such as Grid Search and Randomized Search Cross Validation (CV) were employed, utilizing repeated stratified k fold cross validation to ensure consistent results and reduce the risk of overfitting.

#### K nearest neighbors (KNN)

KNN is a non parametric algorithm used for classification tasks, determining a sample’s class based on the majority vote of its k nearest neighbors. It is particularly effective for datasets with variable distributions and gradual transitions, such as Class 1 (Signal Drift). The hyperparameter k, representing the number of neighbors, was optimized by testing values from 1 to 30. Weighting schemes, including uniform and distance based, and distance metrics like Euclidean, Manhattan, and Minkowski were evaluated. The predicted value is computed as the average of the target values of the k nearest neighbors in Eq. (1):

1 $$\:\widehat{y}=\frac{1}{k}{\sum\:}_{i=1}^{k}{y}_{i}\:$$

where:

$\:\widehat{y}$: predicted value for the target variable.

$$\:k:\:number\:of\:nearest\:neighbors\:considered.$$

$$\:{y}_{i}:value\:of\:the\:target\:variable\:for\:the\:\:{\varvec{i}}^{th}\:nearest\:neighbor.$$

#### Random forest (RF)

Random Forest is a collective learning algorithm that combines multiple decision trees to produce a strong classification by averaging their outputs. It is a very efficient method for handling high dimensional datasets and decreasing the chances of overfitting. This approach is well suited for analyzing positional errors in Class 3 (Localization Inaccuracy). Hyperparameters as the number of trees (n estimators), tree depth, and minimum samples for separations were fine-tuned, with values ranging from 50 to 500 trees, depths between 5 and 20, and separations from 2 to 10. The model’s prediction is provided by:

2 $$\:f\left(x\right)=\frac{1}{N}{\sum\:}_{j=1}^{N}{T}_{j}\left(x\right)$$

Where:

$\:f\left(x\right)\::$ This represents the predicted value for the target variable at data point *x.*

N: This is the total number of decision trees in the Random Forest.

$\:{T}_{j}\left(x\right)$: This represents the prediction of the j th decision tree in the forest for data point *x.*

#### XGBoost

XGBoost stands for advanced Gradient Boosting^40^, an improved boosting algorithm that builds models step by step. The optimization in the performance is reached by the gradient descent approach, which minimizes the prediction error. XGBoost is highly effective in anomaly detection in radio environments because it can handle both linear and nonlinear relationships between anomalies^41^. This controls the model to prevent overfitting and makes it generalize well across new environments. XGBoost makes iteration steps, each round adds tree function to each iteration to reduce the loss. Tree function calculating negative gradient of the loss with respect to the prediction, which can be written as:

3 $$\:\widehat{{y}_{i}^{\left(t+1\right)}}=\widehat{{y}_{i}^{\left(t\right)}}+\eta\:{f}_{t}\left({x}_{i}\right)$$

Where; $\:\widehat{{y}_{i}^{\left(t\right)}}$: is the predicted value in iteration steps.

$$\:{f}_{t}\left({x}_{i}\right)\:\:prediction\:of\:the\:\:weak\:learner\:\left(usually\:a\:tree\right)\:for\:data\:point\:i.$$

$\:\eta\:$ : is learning rate $\:,which\:controls\:the\:step\:size\:of\:the\:update.$

#### Logistic regression (LR)

Logistic Regression is a statistical model for binary classification, predicting probabilities using a sigmoid function. It estimates the probability of an event, such as separating normal (Class 0) from anomalous conditions. Key hyperparameters, including constraint strength (C), solver such as saga, and penalties (L1, L2), were fine tuned. The logistic function is expressed as in Eq. (4):

4 $$\:P\left(y=1|X\right)=\frac{1}{1+{e}^{-\left({{\upbeta\:}}_{0}+{{\upbeta\:}}_{1}{X}_{1}+\dots\:+{{\upbeta\:}}_{n}{X}_{n}\right)}}$$

$\:P\left(y=1|X\right)$: probability of y being 1 given the features X.

*X: input features*.

$\:{\beta\:}_{0}$: This is the intercept term (bias).

$$\:{\beta\:}_{1}:$$

These are the coefficients associated with each feature.

The Logistic Regression equation models the probability of the target variable being 1 as a function of the input features using a sigmoid function (the logistic function). The sigmoid function maps any input value to a value between 0 and 1, which represents the probability.

#### Support vector machine (SVM)

SVM separates classes in high dimensional space by maximizing the gap between them. It is effective for binary classification, such as classifying conditions in Class 5 (LShadLev). Hyperparameter tuning involved exploring kernel alternatives (linear, RBF, polynomial), constraint (C), and kernel coefficients (γ). The decision function is as in Eq. (5)

5 $$\:f\left(x\right)={w}^{T}x+b$$

Where:

$$\:f\left(x\right):is\:the\:decision\:score.$$

$\:{w}^{T}$: *This denotes the transpose of the weight vector.*

*x: This is the input data vector.*

*b: This is the bias term*,* which determines the position of the hyperplane.*

All machine learning models employ the following general tuning strategy:

Randomized Search: originally used to explore rapidly a broad variety of hyperparameters.

Grid Search: Applied for fine tuning after limiting promising parameter ranges.

Cross Validation: Repeated stratified k fold with K = 5, was used to guarantee reliable performance metrics and prevent data leakage.

Performance Metrics: Metrics like accuracy, precision, recall, F1 score, and AUC ROC were monitored to detect the best hyperparameter combinations for each model.

## Results and discussion

This section serves as a foundation for understanding experimental findings and drawing meaningful ideas. The performance of the classification model is evaluated using four main parameters: accuracy, recall, F1 score, and accuracy. Their definitions and synonyms are as follows:

Accuracy: This indicates how many predicted positive cases are actually correct. Its formula is given by.

6 $$Accuracy=\frac{{T}_{P}+{T}_{n}}{Total\:samples}$$

where, $\:{T}_{n}$ refers to true negatives and $\:{T}_{P}$ refers to true positive

Precision: This measures how many of the instances predicted as positive are actually correct. It is calculated as follows:

7 $$\:\text{P}\text{r}\text{e}\text{c}\text{i}\text{s}\text{i}\text{o}\text{n}=\frac{{T}_{P}}{{T}_{P}+{F}_{P}}$$

Recall: This indicates how many of the actual positive instances were correctly identified by the model.

8 $$\:\text{R}\text{e}\text{c}\text{a}\text{l}\text{l}=\frac{{T}_{P}}{{T}_{P}+{F}_{n}}$$

F1 Score: A balanced metric that combines Precision and Recall, giving a single score that reflects both. It is computed using the following formula:

9 $$\:\text{F}1\:\text{S}\text{c}\text{o}\text{r}\text{e}=\frac{{T}_{P}+{T}_{n}}{{T}_{P}+{T}_{n}+{F}_{P}+{F}_{n}}$$

Where $\:{F}_{P}\:is\:false\:positive,\:{F}_{n}$ signifies false negatives, $\:{T}_{n}$​ refers to true negatives and $\:{T}_{P}$ refers to true positive

**Fig. 6:**
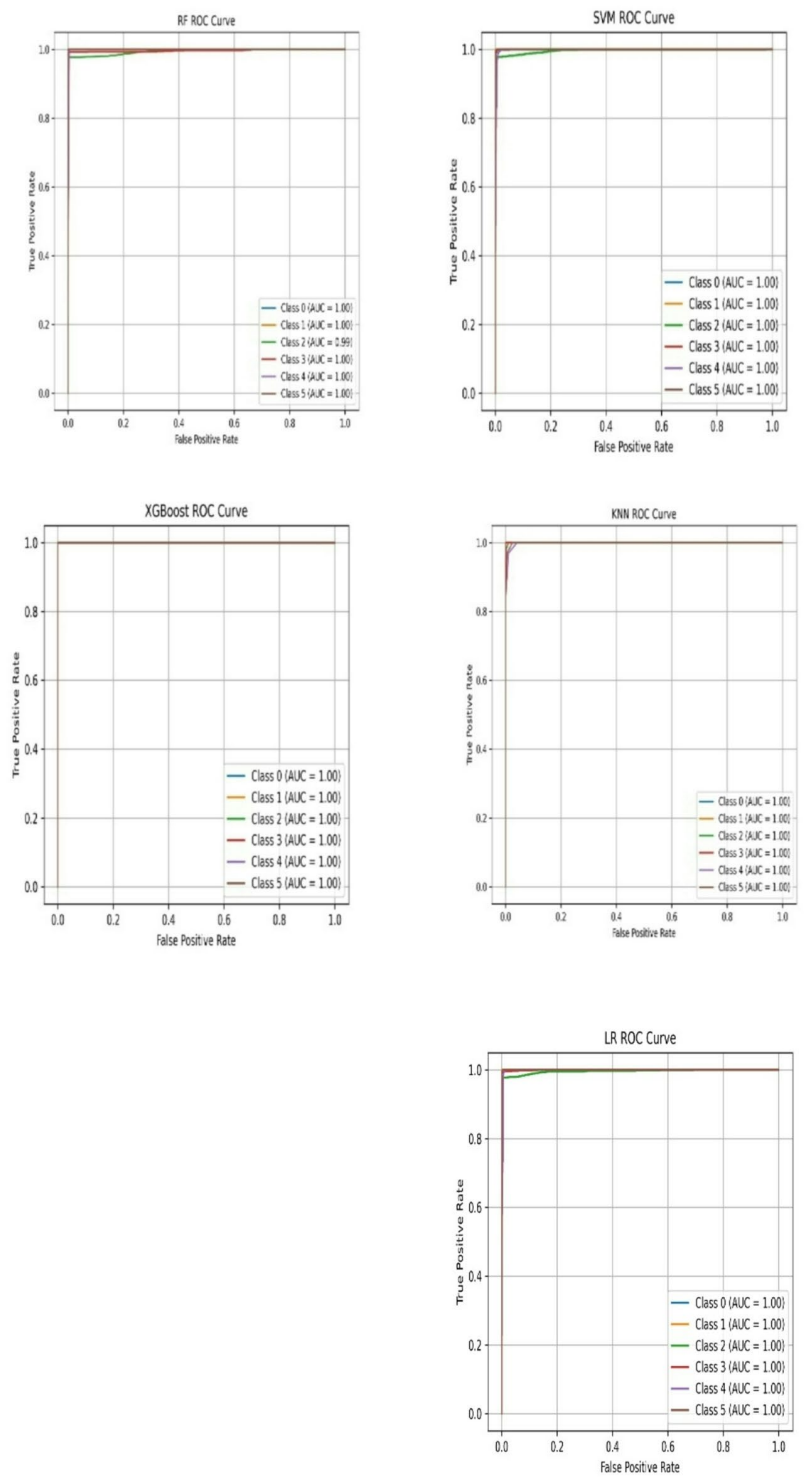
ROC curves for the machine learning Models.

Figure 6 displays the Receiver Operating Characteristic (ROC) curves for five different machine learning models used to detect anomalies in wireless networks, highlighting their classification performance across six network states. The Random Forest classifier excels, with an Area Under Curve (AUC) of 1.00 for Classes 0, 1, 3, 4, and 5, though Class 2 (Multipath Effect) shows a slight performance drop in results with AUC of 0.98. The Logistic Regression model also achieves perfect classification, with AUC scores of 1.00 across all classes, marked by sharp vertical lines that indicate very few false positives.

In this study, the misclassification between the HShadLev and LShadLev classes is important point to make classification. These two scenarios were difficult to distinguish because their signal characteristics often overlapped. In environments with high shadowing, significant signal attenuation can occur, but it may resemble normal path loss depending on the conditions, making it hard to identify as an anomaly. On the other hand, low shadowing levels cause minimal distortion, making it equally challenging for the model to classify these instances as anomalies. The difficulty arose from feature overlap metrics such as RSSI, Path Loss, and SNR values in these cases did not significantly change from normal conditions, leading to misclassifications. To resolve this, we applied advanced feature engineering techniques, including time series analysis to monitor the temporal changes in signal degradation. Additionally, we enhanced the model by combining Random Forest with anomaly specific boosting methods, which allowed for better differentiation between HShadLev and LShadLev, significantly improving the accuracy of these classifications.

**Fig. 7:**
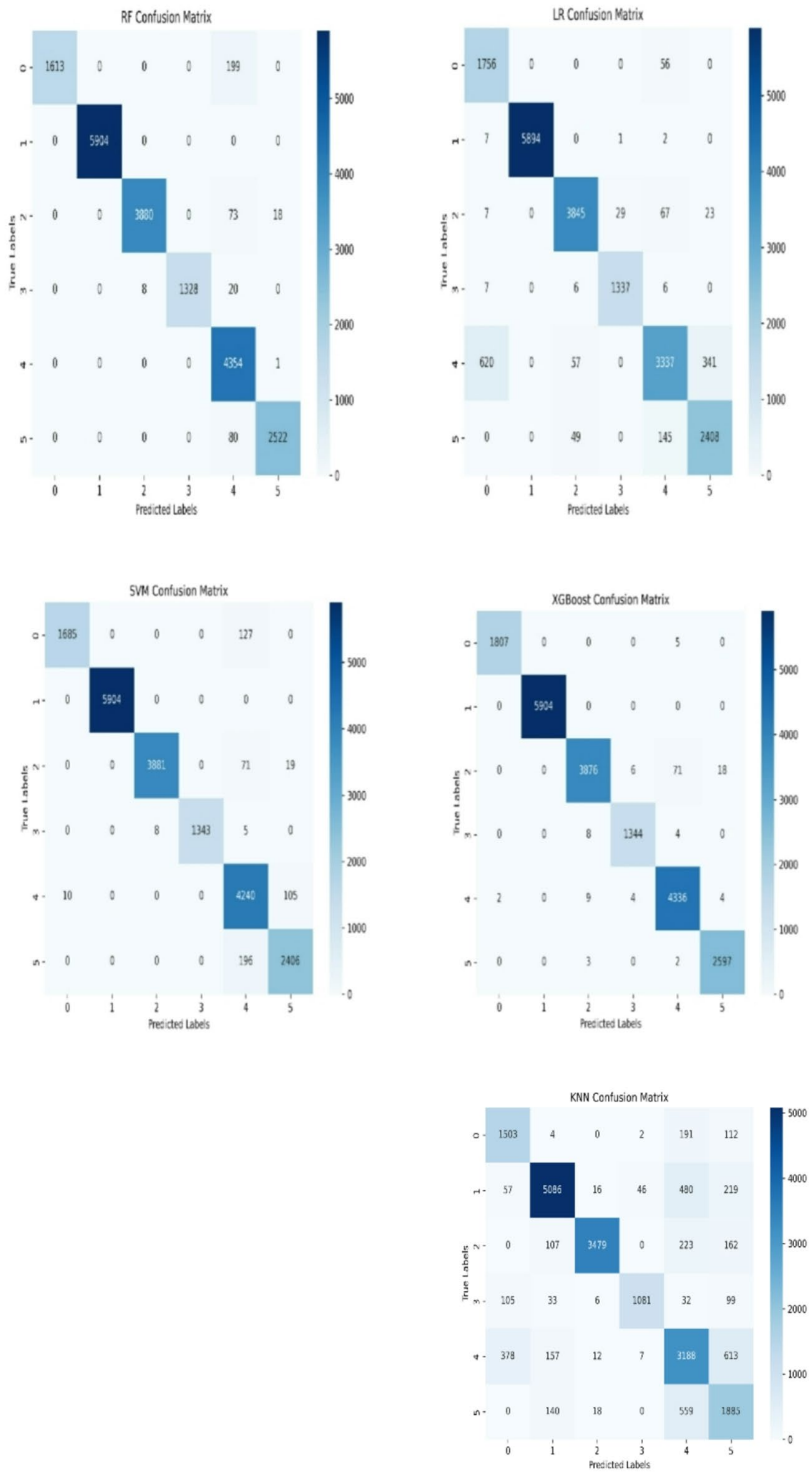
Confusion matrix for the machine learning models.

Figure 7 displays the confusion matrices for five machine learning models used in wireless network anomaly detection, showing how well each model performs across six network states (0–5). The Random Forest classifier shows strong performance, with most of its predictions falling on the diagonal, indicating correct classifications. However, there are a few misclassifications between Classes 4 and 5. The Logistic Regression model also performs well, with 5,894 correct classifications for Class 1 (Signal Drift) and 2,408 for Class 5 (LShadLev). However, it has some misclassifications, especially between Class 4 and Classes 0 and 5, with 620 and 341 misclassified instances, respectively. The Support Vector Machine (SVM) model performs excellently, with 5,904 correct predictions for Class 1 and 4,240 for Class 4 (HShadLev). There’s only a few amounts of confusion between Classes 4 and 5, with 105 instances misclassified. The XGBoost model also shows strong accuracy, with 5,904 correct predictions for Class 1 and 4,336 for Class 4, and only few misclassifications across different classes.

**Fig. 8:**
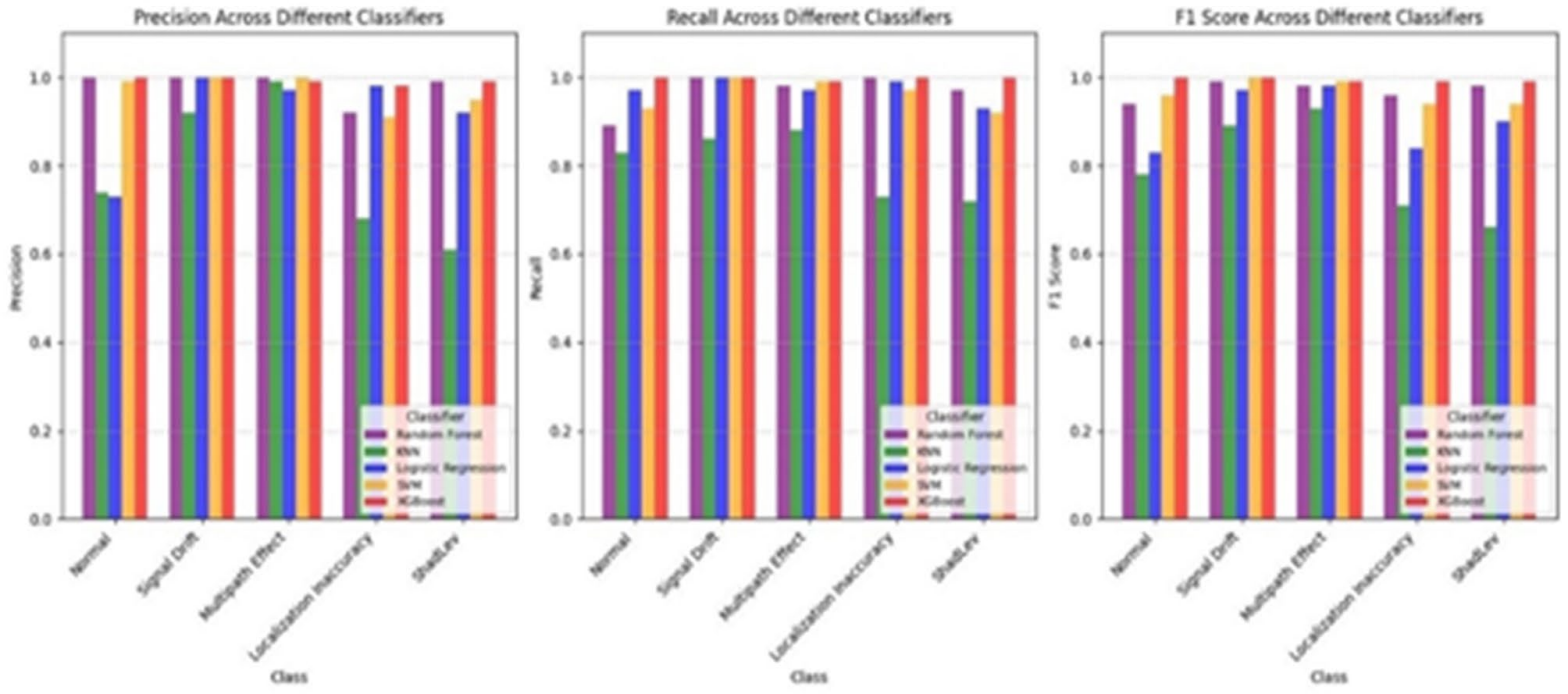
Evaluation metrics based on machine learning models.

Figure 8 shows the performance comparison of five machine learning classifiers: RF, KNN, LR, SVM, and XGBoost. The assessment covers six classes: Normal, Signal Drift, Multipath Effect, Localization Inaccuracy, and ShadeLv, using three key metrics: Precision, Recall, and F1 Score.

XGBoost and SVM produced high precision across all classes, ranging from 0.95 to 1.0. Random Forest obtains precision ≥ 0.98 for Signal Drift, Multipath Effect, and ShadeLv but drops to ~ 0.9 for Localization Inaccuracy. Logistic Regression maintains 0.95 precision for most classes but falls to 0.7 for Normal and 0.85 for Localization Inaccuracy. KNN has the highest variance, with 0.75 for Normal and 0.6–0.7 for ShadeLv and Localization Inaccuracy.

XGBoost, SVM, and RF achieved 0.95 across most classes. SVM and XGBoost reach 1.0 recall for Signal Drift and Multipath Effect. LR records 0.85 recall for Normal and ~ 0.9–0.95 for other classes. KNN has the minimal recall, with 0.7 for Normal and 0.6 for Localization Inaccuracy.

XGBoost and SVM maintain F1 Scores between 0.95 and 1.0 across all classes. RF achieves with 0.98 F1 Score for most classes, with a decrease 0.9 for Localization Inaccuracy. Logistic Regression records an F1 Score of 0.85 for Normal, ~ 0.9 for Localization Inaccuracy, and 0.95 for other classes. KNN has the lowest F1 Scores (~ 0.6 for Localization Inaccuracy and ~ 0.7 for ShadeLv).

Table 4 presents a comprehensive comparative analysis of five machine learning models RF, KNN, LR, (SVM), and XGBoost employed for anomaly detection in wireless networks. The evaluation metrics encompass Precision, Recall, and F1 Score for six distinct classes of network behavior, along with the overall accuracy for each model. The XGBoost model demonstrates superior performance with the highest overall accuracy of 0.99, achieving perfect scores (1.00) for normal traffic and signal drift detection, while maintaining extremely high performance (0.98-1.00) across all other anomaly classes. Right after, the RF classifier exhibits robust performance with an overall accuracy of 0.98, showing perfect precision (1.00) for the first four classes and maintaining high performance (> 0.92) for higher level shadow fading scenarios.

The SVM model achieves an impressive overall accuracy of 0.97, with particularly strong performance in detecting signal drift and localization inaccuracies (F1 scores of 1.00). The LR model maintains steady performance with an overall accuracy of 0.93, though showing some variation in its handling of normal traffic (precision: 0.73, recall: 0.97) and HShadLev anomalies (precision: 0.92, recall: 0.77).

The KNN model, while achieving an overall accuracy of 0.81, demonstrates relatively lower performance compared to other models, especially in detecting HShadLev and LShadLev anomalies (F1 scores of 0.71 and 0.66 respectively). However, it maintains reasonable performance for multipath effect detection with an F1 score of 0.93. Notably, all models perform exceptionally well in detecting signal drift (Class 1), with F1 scores ranging from 0.89 to 1.00. The detection of shadow fading effects (Classes 4 and 5) proves more challenging across all models, though XGBoost and Random Forest maintain high accuracy even for these complex scenarios.

**Table 4:** Evaluation metrics report for all machine learning models.

Class	Precision	Recall	F1 Score
Random forest evaluation metrics report Overall accuracy: 0.98			
0 (Normal)	1.00	0.89	0.94
1 (Signal Drift)	1.00	1.00	1.00
2 (Multipath Effect)	1.00	0.98	0.99
3 (Localization Inaccuracy)	1.00	0.98	0.99
4 (HShadLev)	0.92	1.00	0.96
5 (LShadLev)	0.99	0.97	0.98
KNN evaluation metrics report Overall accuracy: 0.81			
0 (Normal)	0.74	0.83	0.78
1 (Signal Drift)	0.92	0.86	0.89
2 (Multipath Effect)	0.99	0.88	0.93
3 (Localization Inaccuracy)	0.95	0.80	0.87
4 (HShadLev)	0.68	0.73	0.71
5 (LShadLev)	0.61	0.72	0.66
Logistic regression evaluation metrics report Overall accuracy: 0.93			
0 (Normal)	0.73	0.97	0.83
1 (Signal Drift)	1.00	1.00	1.00
2 (Multipath Effect)	0.97	0.97	0.97
3 (Localization Inaccuracy)	0.98	0.99	0.98
4 (HShadLev)	0.92	0.77	0.84
5 (LShadLev)	0.87	0.93	0.90
SVM evaluation metrics report Overall accuracy: 0.97			
0 (Normal)	0.99	0.93	0.96
1 (Signal Drift)	1.00	1.00	1.00
2 (Multipath Effect)	1.00	0.98	0.99
3 (Localization Inaccuracy)	1.00	0.99	1.00
4 (HShadLev)	0.91	0.97	0.94
5 (LShadLev)	0.95	0.92	0.94
XGBoost evaluation metrics report Overall accuracy: 0.99			
0 (Normal)	1.00	1.00	1.00
1 (Signal Drift)	1.00	1.00	1.00
2 (Multipath Effect)	0.99	0.98	0.99
3 (Localization Inaccuracy)	0.99	0.99	0.99
4 (HShadLev)	0.98	1.00	0.99
5 (LShadLev)	0.99	1.00	0.99

As shown in Table 5, the comparison highlights the strengths, limitations, and applications of our proposed DT framework for anomaly detection in wireless networks compared to previous systems. Previous methodologies, such as preprocessing for CNN models^2^, DT based anomaly detection^4^, and both supervised and unsupervised anomaly detection techniques^7^, achieved notable outcomes in their respective domains. For instance, the CNN model in^2^ reached 100% testing accuracy for underwater communications, while the DT approach in^4^ successfully generated 20,000 samples without hyperparameter tuning. However, these methods exhibit limitations, such as sensitivity to noise, reliance on basic algorithms, and the need for extensive training datasets. Similarly, advanced reviews and implementations like^14,22^ address broader challenges, including scalability and data quality, yet face issues with limited sources and false positives, which reduce their effectiveness. Our proposed system builds on these ideas by combining DT technology with ensemble machine learning methods, offering scalability and improved anomaly detection.

The results of our proposed system demonstrate a significant advancement over existing solutions. By fine tuning XGBoost models within the DT framework, our approach excels in identifying and mitigating wireless anomalies, ensuring resilience against threats such as jamming attacks. Unlike prior systems, our work integrates cutting edge methodologies to improve scalability, monitoring accuracy, and network security. Nevertheless, as noted in Table 5, the primary weakness of our system lies in its delayed deployment due to the unavailability of USRP units required for practical validation. Once the hardware is integrated, our framework will be fully implemented and tested, enabling comprehensive performance evaluation in real world scenarios. This ongoing work underscores the potential of our proposed system to address critical gaps in anomaly detection and security risk analysis for DT wireless networks.

**Table 5:** Comparative analysis between our work and previous work.

Refs.#	Methodology	Objective	Key Results	Weakness	Applications
^2^	Preprocesses data for effective training of the CNN model	Deep learning used to updates underwater communication accuracy.	The CNN model attained 100% testing accuracy.	Conventional methods are noise sensitive; current methods lack robustness.	Water quality monitoring, underwater exploration, mapping.
^3,4^	DT structure, anomaly detection, comparison.	Novel anomaly detection and DT, simulations.	20,000 samples generated, no hyperparameter tuning.	Basic and advanced algorithms are used to check the performance.	Anomaly detection in cellular networks based on DT technology.
^7^	Supervised and unsupervised techniques used to detect anomalies.	Classify anomalies, develop detection models.	High F1 scores accomplished for anomalies (1.0to0.98).	ML techniques require sufficient data for training and validation.	Automatic detection of radio environment anomalies in IoT.
^14^	Use six Deep Learning techniques, intrusion detection.	Study evaluates ML techniques for anomaly detection efficiency.	290 studies on ML, selection based on best quality criteria.	limited sources with strict criteria.	Detection techniques for span networks, sensors, medical, and power systems.
^22^	Clustering, density, PCA, autoencoders, isolation forest, hybrid anomaly detection.	Review approaches, improve scalability, address challenges, explore hybrids, discuss ethics.	Unsupervised approaches, challenges, scalability, hybrid models.	Unsupervised learning suffers with quality, drift, dimensions, labels, false positives.	Healthcare monitoring, cybersecurity, industrial IoT anomaly detection methods.
^26^	DTs, detection models, hybrid techniques, compressed sensing, data assimilation.	Discover challenges, technologies, implications, values, and history of DTs.	Hybrid between physics based and data driven models.	DTs demand accuracy, scalability, security, seamless integration, and adaptability.	IoT facilitates real time health, maintenance, and agriculture solutions.
^30^	Bayesian control, variational inference, multi agent systems, uncertainty quantification.	Create Bayesian DTs, uncertainty, control, optimization, and validation.	Mean field variational inference for approximating posterior distributions.	Model limitations, data quality, and computational complexity challenges affect decisions.	MARL control, anomaly detection, uncertainty prediction, optimized data collection.
^33^	ML algorithms, ray tracing, clustering, interpolation, calibration for radio environments modeling.	Explore ML advancements, challenges, accuracy, and research in wireless systems.	ML improves propagation in urban areas, RSRP prediction, tower localization.	Costly data collection, environmental effects, crowd sourcing limitations.	Path loss estimation, radio mapping, tower localization.
The proposed work	Anomaly detection based on DTs with ML models, ensemble methods.	Create scalable DTs framework for resilient anomaly and security detection.	XGBoost excelled, fine tuning improved anomaly detection with DTs.	Its lack of validation and deployment in real world scenarios, pending the integration of Raspberry Pi as central unit required for practical implementation	DTs technology enables wireless anomaly detection, monitoring, and resilience against threats like signal drift and jamming attack.

## Conclusion

This work compares machine learning models for anomaly detection in wireless communication networks based on performance measures like Precision, Recall, F1 Score, and overall accuracy for different classes of network behavior. The comparison demonstrates high performance of ensemble methods and specifically XGBoost and Random Forest with the highest accuracy. XGBoost performs well with an accuracy of 0.99, having almost perfect detection across all anomaly classes, including normal traffic and signal drift, and thus is highly reliable solution for network security. Random Forest follows closely with an accuracy of 0.98, excelling in detecting most anomaly types while effectively handling challenging shadow fading scenarios.

The SVM model achieves notable accuracy (0.97) and excels in identifying signal drift and localization errors, showcasing its reliability for specific anomaly classes. Logistic Regression, with an overall accuracy of 0.93, performs well but shows variability in detecting normal traffic and shadow fading anomalies. The KNN model, while achieving a lower accuracy of 0.81 compared to other models, remains effective in detecting multipath effects but struggles with shadow fading classes. Across all models, detecting signal drift proves to be the most consistent, while shadow fading anomalies present greater challenges, emphasizing the complexity of these scenarios.

The results demonstrate that ensemble methods, such as XGBoost and Random Forest, consistently outperform traditional classification techniques in wireless network anomaly detection. These findings highlight the importance of adopting robust, fine tuned models that can handle diverse network behaviors without needing complex learning models, particularly the use of Raspberry Pi doesn’t need any complex model to fit with real time data and make it easy to detect the anomalies.

This research demonstrates a current integration of machine learning models with modern anomaly detection frameworks in order to assure the reliability and security level of wireless networks. With its comprehensive datasets and advanced analytics, the offered methodology here provides wider anomalous situations coverage, where accuracy and timeliness guarantee proper network issues detection. Future work will focus on expanding the dataset with more environmental factors and types of attacks, which will also investigate deep learning techniques to further improve detection capabilities. Eventually, this research contributes to developing intelligent and adaptive systems that will be an intrinsic part of securing evolving wireless technologies, including 5G and industrial IoT environments.

This study uses machine learning models for anomaly detection because of their simplicity and effectiveness in identifying patterns in wireless communication networks. However, the current approach relies on batch processing, meaning it does not detect anomalies in real time. In dynamic and time sensitive environments like industrial IoT and 6G networks, real time detection is essential for immediate response and system reliability. Future work will focus on adapting the framework to process data in real time by integrating streaming data techniques, online learning models, and edge computing. Implementing adaptive thresholding will also help minimize detection delays while maintaining accuracy. These improvements will make the system more responsive and practical for real world deployment, ensuring it can handle evolving wireless communication challenges efficiently.

Our objective in the next stage is to strike a balance between efficiency and accuracy, ensuring a robust and deployable solution without overcomplicating the model. This is especially important as we are implementing real time monitoring and utilizing ensemble models on Raspberry Pi act as central unit to ensure the concept of Digital twin.

## Data Availability

The datasets analyzed during the current study are available from the corresponding author on reasonable request.
